# Expression Analysis of Muscle-Specific miRNAs in Plasma-Derived Extracellular Vesicles from Patients with Chronic Obstructive Pulmonary Disease

**DOI:** 10.3390/diagnostics10070502

**Published:** 2020-07-21

**Authors:** Sara Carpi, Beatrice Polini, Dario Nieri, Nevio Dubbini, Alessandro Celi, Paola Nieri, Tommaso Neri

**Affiliations:** 1 Department of Pharmacy, University of Pisa, via Bonanno 6, 56126 Pisa, Italy; beatrice.polini@farm.unipi.it (B.P.); paola.nieri@unipi.it (P.N.); 2 Centro Dipartimentale di Biologia Cellulare Cardiorespiratoria, Dipartimento di Patologia Chirurgica, Medica, Molecolare e di Area Critica e Azienda Ospedaliero-Universitaria Pisana, 56126 Pisa, Italy; darionieri@hotmail.it (D.N.); alessandro.celi@unipi.it (A.C.); tommaso.neri79@for.unipi.it (T.N.); 3 Miningful Studio; neviod@miningfulstudio.eu

**Keywords:** chronic obstructive pulmonary disease, COPD, microRNA, extracellular vesicle

## Abstract

MicroRNAs (miRNAs) are a class of short non-coding RNAs involved in the regulation of gene expression and the control of several cellular processes at physiological and pathological levels. Furthermore, extracellular vesicles (EV), which are small membrane-bound vesicles secreted by cells in the extracellular environment, contain functional miRNAs. The remarkable deregulation of many miRNAs has been demonstrated in respiratory diseases. Among them, miR-206, miR-133a-5p, and miR-133a-3p are striated muscle-specific miRNAs (myo-miRNA), related to skeletal muscle dysfunction, one of the commonest systemic manifestations in patients with chronic obstructive pulmonary disease (COPD). Nevertheless, their circulating expression in COPD patients is not demonstrated. For these reasons, we performed a pilot study to analyze the expression profiles of myo-miRNAs in plasma-derived EV from patients with COPD. We analyzed the expression profiles of selected myo-miRNAs in plasma-derived EV from COPD. Receiver operating characteristic analyses were carried out to evaluate whether selected plasma miRNAs were able to discriminate between different groups of COPD patients. We found EV-embedded myo-miRNAs in the bloodstream of COPD patients. Specifically, miR-206, miR-133a-5p and miR-133a-3p were significantly upregulated in group B patients. Receiver operating characteristic analyses of the combination of these selected miRNAs showed their high capacity to discriminate group B from other COPD patients. Our data provide evidence that myo-miRNA are present in EV in the plasma of COPD patients and their expression (miR-206, miR-133a-5p, and miR-133a-3p) can discriminate group B from group C patients. The future analysis of a larger number of patients should allow us to obtain more refined correlations.

## 1. Introduction

Chronic obstructive pulmonary disease (COPD) is a heterogeneous and complex disease associated with significant morbidity and mortality and represents a leading cause of mortality worldwide [1]. Over the last ten years, the Global Initiative for Chronic Obstructive Lung Disease (GOLD) document has moved beyond a lung function-centered view (only based on Forced Expiratory Volume in the first second (FEV1)) towards a more comprehensive approach, also including exacerbation history and symptoms. Accordingly, since the 2011 version four groups have been identified (named from A to D) [2], while group A contains the mildest patients and group D, conversely, the most severe ones, establishing disease severity among groups B and C is still challenging [3]. These groups, however, are still considered unsatisfactory to fully classify the disease for clinical and research purposes [4,5,6], since COPD represents a complex pathological condition with several important extrapulmonary manifestations (cardiovascular diseases, skeletal muscle dysfunction and subsequent deconditioning, sarcopenia, metabolic disorders, etc.) [2]. Indeed, current research in the COPD field is focused on the identification of biomolecules, each accounting for a specific pathobiological pattern of disease (called the endotype) [7].

Since it is not feasible to obtain lung tissue samples from patients in clinical practice, it is not easy to obtain pathological information on the pulmonary state. Hence, biofluids such as blood, bronchoalveolar lavage fluid, and sputum represent important sources of circulating biomolecules able to give information about lung conditions.

In this context, circulating microRNAs (miRNAs) and, particularly, miRNA embedded in extracellular vesicles (EV) are gaining a growing interest in the area of respiratory medicine [8].

EV are submicron (100–1000 nm) vesicles shed by cells during activation and early apoptosis [9]. EV are found in different types of biofluids where they likely play a role in intercellular communication in a variety of pathophysiological conditions, such as inflammation and blood coagulation, and are involved in numerous lung diseases [10]. They have been shown to carry and transfer a wide variety of molecules, such as proteins and nucleic acids, including miRNAs, as mediators of intracellular communication.

MiRNAs are short non-coding RNAs able to induce a pleiotropic modulation of gene expression and a wide spectrum of biological processes. Currently, more than two thousand miRNAs have been discovered in humans, and their information is reported in miRBase, a public and free database available at [5,6,7]. 

Many miRNAs are ubiquitously expressed in every cell type, but some are tissue specific when the miRNA expression is 20-fold higher than the mean expression in other tissues [11]. MiR-206, miR-133a-3p, miR-133a-5p and others represent a sub-group of striated muscle-specific miRNAs named myo-miRNAs. Myo-miRNAs are involved in skeletal muscle myogenesis, differentiation and the specification of fiber type, skeletal muscle stem cells and muscle regeneration [12].

Interestingly, circulating miRNAs are stable in biofluids and, after extraction [13], they can be profiled by relatively simple methods, i.e., quantitative real-time PCR (qRT-PCR), microarray or sequencing technology [14]. Therefore, miRNAs carried in EV have the potential to become clinical biomarkers [9].

Among miRNAs, myo-miR caught our attention because their expression in EV isolated from COPD patients is unexplored, despite the evidence that they play a crucial role in the control of skeletal muscle function [6,15,16] and that skeletal muscle dysfunction is one of the commonest systemic manifestations in many COPD patients [17,18,19].

For these reasons, we conducted a pilot study to analyze the expression profiles of EV-derived myo-miRNAs (specifically miR-206, miR-133a-5p, and miR-133a-3p) in plasma samples collected from patients with COPD; in particular, we tried to understand if these myo-miRNAs could help discriminate between patients arbitrary classified according to the GOLD 2011 document, while also considering the biological processes known to be associated to these myo-miRNAs through gene ontology analysis. Furthermore, we also analyzed EV counts in the four COPD groups.

## 2. Materials and Methods

### 2.1. Study Cohort Characteristics

We enrolled consecutive patients with COPD, diagnosed according to the GOLD document, with post-bronchodilator Forced Expiratory Volume in the 1st second (FEV1)/Forced Vital Capacity <0.7 and FEV1 between 30 and 70% of the predicted value. The main exclusion criteria were: history of asthma; COPD exacerbation(s) in the 6 weeks preceding enrolment; acute or chronic respiratory failure; severe heart failure; recent (≤3 months before the study) acute coronary syndrome, pulmonary embolism or major surgery; active cancer. All patients were recruited among those regularly attending the outpatient service of the Respiratory Pathophysiology and Rehabilitation Unit of Pisa University Hospital. For all patients, we collected clinical history and symptoms score (both according to the modified Medical Research Council (mMRC) scale and COPD Assessment Test (CAT^2^)) and obtained pulmonary function tests, with a bronchodilator test performed after a 24-h inhaled treatment withdrawal.

Patients were grouped by GOLD 2011 classification into 4 groups (from A to D) according to FEV1 values, symptoms (baseline mMRC and CAT), and exacerbation history [2].

The current study was approved by the local Ethics Committee (approval code n. 1088, 15/06/2016)), in compliance with the Declaration of Helsinki, and all participants signed written informed consent forms.

### 2.2. Isolation and Characterization of Extracellular Vesicles

Circulating EVs were obtained from 4 mL of peripheral blood as previously described [20]. For the analysis of miRNAs contained in EV, we isolated the EVs from platelet-poor plasma. Briefly, blood was drawn into sodium citrate. Platelet-poor plasma (PPP) was obtained by two subsequent centrifugations: 1500× *g* for 15 min and 13,000× *g* for 2 min at room temperature. PPP was then centrifuged at 16,000× *g* for 45 min to obtain EV pellets. EV pellets were stored at −80 °C and subsequently used for EV analysis. Multiparametric flow cytometry analysis was performed on a Beckton Dickinson FACS-CANTO flow cytometer. The following gating strategy was used: events were first analyzed for their physical parameters (forward (F) vs. side (S) scatter (Sc)); a blend of fluorescent beads of three diameters (0.5, 0.9 and 3 μm) was used to identify them and to standardize the protocol. Annexin-V labeled with peridinin chlorophyll protein complex was used as a general marker for phosphatidylserine-exposing EV. Values are reported as events with the same forward scatter (FSC) /side scatter (SSC) parameters of the 0.5–0.9 μm beads and annexin-V recorded in a total observation time of 5 min in low flow conditions [21]. Repeated measurements were obtained on different samples, randomly selected within both patients and control subjects, and good repeatability was observed.

### 2.3. Evaluation of EV-Derived miRNA Expression

The miRNeasy Micro Kit (Qiagen, Hilden, Germany) was used for the purification and extraction of miRNAs from EV isolated from the plasma of COPD patients. The retro-transcription and qPCR experiments of extracted miRNAs were performed as previously reported [22]. MiScript Primer Assays specific for hsa-miR-206 (MIMAT0000462), hsa-miR-133a-5p (MIMAT0026478) and hsa-miR-133a-3p.2 (MIMAT0000427) were used. MiRNA expression was calculated using the Delta threshold cycle (Ct) method and normalized to *Caenorhabditis elegans* miR-39 (*Cel-miR*-39). The value reported on the *y*-axis in relative figure is the fold change, determined by the comparative Ct method, using *Cel*-miR-39 as internal control.

### 2.4. miRNA Target Prediction and Pathway Analysis

The top predicted targets from TargetScan (Human version 7.2) were subjected to computational analysis with the database for annotation, visualization, and integrated discovery (DAVID) tool (version 6.8) [23] to identify biological pathways associated with the miRNAs modulated in patients with COPD.

### 2.5. Statistical Analysis 

The statistical significance of miRNA levels was analyzed by a Kruskal–Wallis test followed by Dunn’s multiple comparisons (GraphPad Prism 8). Linear regression followed by receiver operating characteristics (ROC) analyses were carried out to evaluate whether the selected miRNAs were able to discriminate between different COPD groups. The statistical significance of single-miRNA ROC curves was tested by fitting a univariate logistic regression model. The panels were built and tested by fitting a multivariable binomial generalized linear model, with a logit link function. All statistical analyses were performed using R, the R Project for Statistical Computing software package, version 3.6.0, with a significance level of 0.05.

## 3. Results

### 3.1. Patient Characteristics and Enumeration of EV According to GOLD Groups

Blood samples were collected and EV isolated from 35 COPD patients with different disease severities, classified according to GOLD 2011 guidelines [2] into four groups (from A to D). All patients were current or former smokers, with moderate to severe airflow obstruction (mean FEV1 53.9% predicted). Group A was poorly represented (only four subjects, as expected, since these less symptomatic patients are known to rarely seek specialist consultation), while group D patients represented about 40% of the whole sample. The main anthropometric and functional data of the study population are reported in Table 1. Furthermore, we subsequently divided the whole population sample into four groups (from A to D) according to the 2011 GOLD guidelines [2]. as expected, we found significant differences only among FEV1 values and symptom scores (evaluated both with the modified Medical Research Council scale and COPD Assessment Test) (see Table 1).

**Figure 1:**
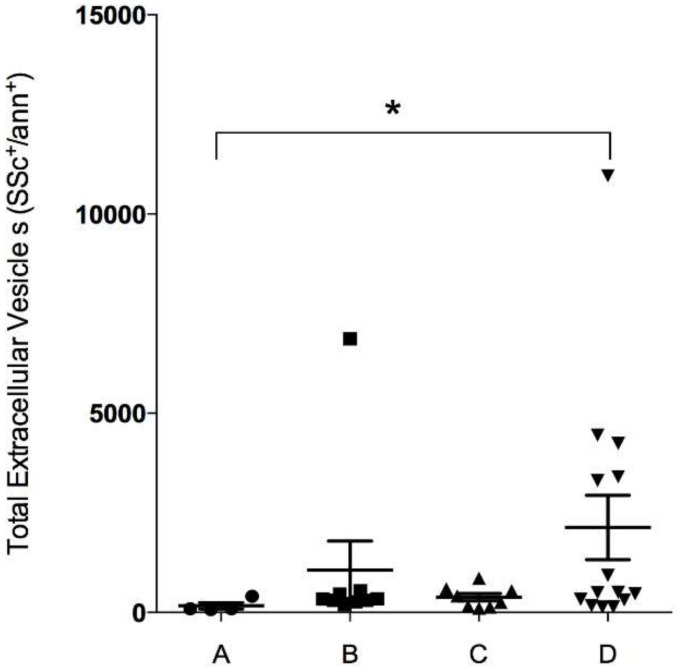
Flow cytometry analysis of total extracellular vesicles (EV) in plasma of chronic obstructive pulmonary disease (COPD) patients. Ordinary one-way ANOVA followed by Kruskal–Wallis multiple comparisons test (* *p* < 0.05).

We analyzed total EV in the four groups of COPD patients, and found a significant increase in total EV in group D vs. group A and an increasing trend in group C vs. group A, as shown in Figure 1.

### 3.2. Muscle-Specific miRNAs Expression in Plasma-Derived EV

To study muscle-specific miRNA expression in the EV components of plasma from patients with COPD, we analyzed and detected the expression of three myo-miRNAs (miR-206, miR-133a-5p, and miR-133a-3p).

**Figure 2:**
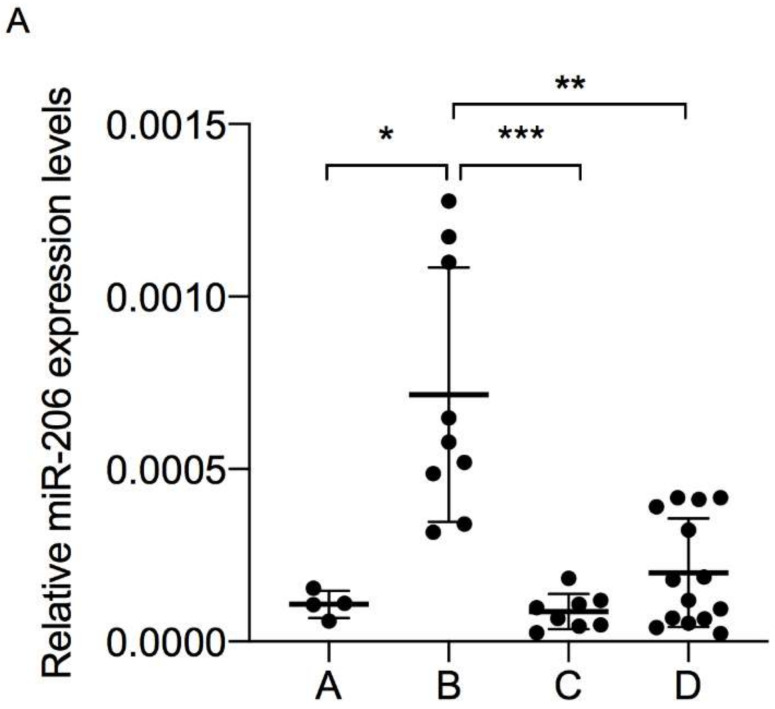

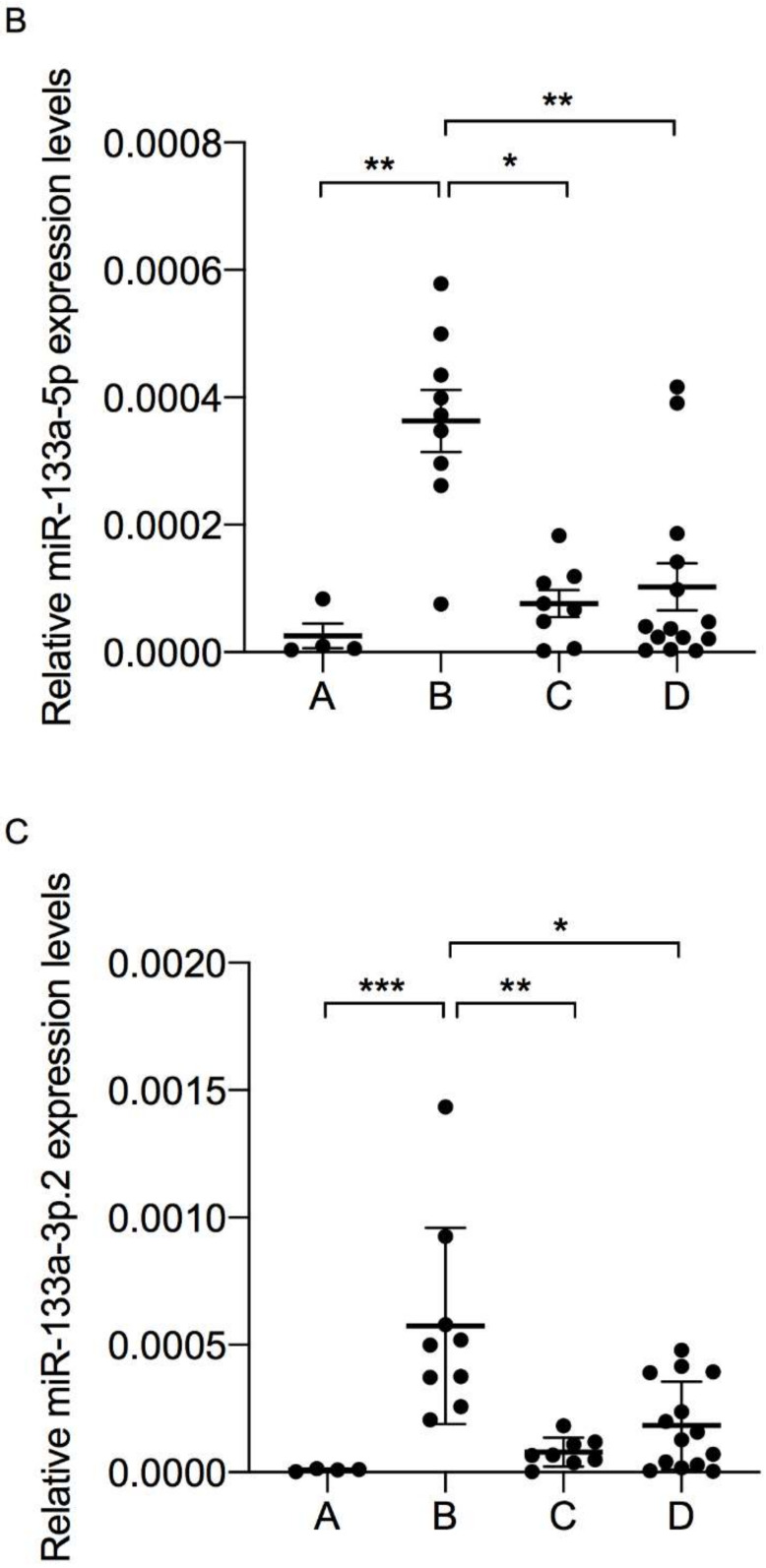
Total microRNAs were extracted from extracellular vesicles plasma-derived of chronic obstructive pulmonary disease (COPD) patients, and miR-206 (**A**), miR-133a-5p (**B**), and miR-133a-3p.2 (**C**) levels were measured by qPCR, analysis using the Ct method, and normalized to Cel-miR-39. Kruskal–Wallis followed by Dunn’s multiple comparisons test (* *p* < 0.05, ** *p* < 0.01, *** *p* < 0.001).

The three myo-miRNAs analyzed were modulated in relation to COPD groups, highlighting an interesting trend, especially the group B COPD patients. Indeed, EV-derived miR-206, miR-133a-5p and miR-133a-3p (which we called “triple signature”) were significantly (*p* < 0.05) upregulated in group B compared to A, C and D (Figure 2).

No significant myo-miRNA deregulation due to sex, age, EV numbers or any clinical-pathological factors (including current inhaled therapy at enrolment) was observed (data not shown).

### 3.3. Classification Value of Triple Signature of EV-Derived myo-miRNAs

The statistical significance of the EV-derived myo-miRNA measurements was evaluated by receiver operating characteristics (ROC) analysis, obtained by plotting the rate of true positives (sensitivity) versus false positives (1-specificity) (Table 2 and Figure S1).

The results show that the triple signature can discriminate GOLD group B from C with high specificity (area under the curve (AUC) of 89.74%), but displays an acceptable score even versus A and D (AUC of 83.9% and 69.4%, respectively). Notably, the triple signature showed a very high statistical significance in terms of its capability to distinguish GOLD group B from the others.

### 3.4. Biological Processes Associated with myo-miRNAs Modulation

By using the database for annotation, visualization and integrated discovery (DAVID) tool, we performed an analysis of the biological processes associated with the genes potentially altered in patients in group B of COPD: 3970 mRNA genes were associated with the three upregulated myo-miRNAs. 

**Figure 3:**
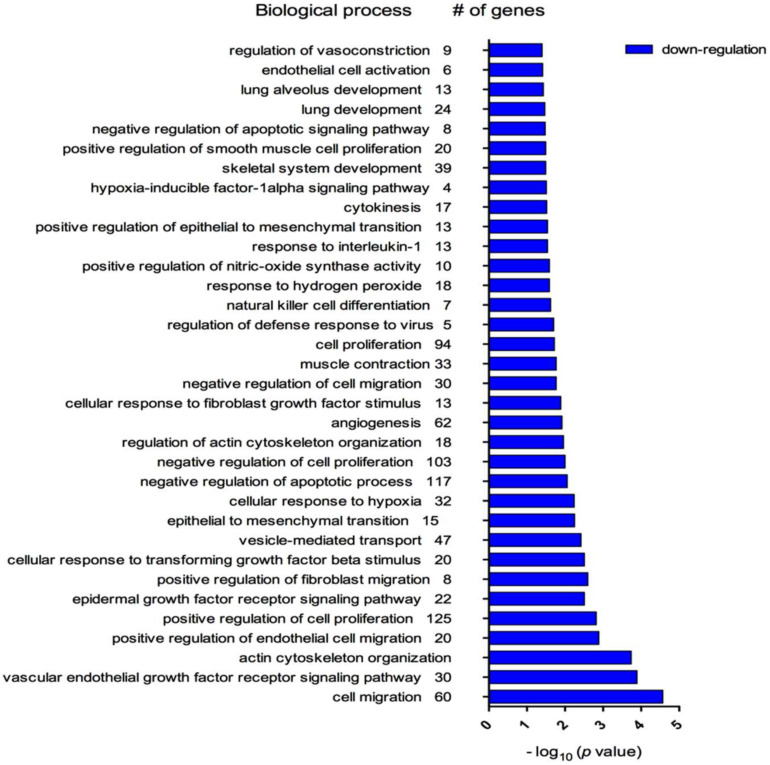
Biological processes, obtained by database for annotation, visualization, and integrated discovery (DAVID) analysis, associated with target genes of striated muscle-specific microRNAs from plasma-derived extracellular vesicles characterizing patients with chronic obstructive pulmonary disease (COPD), group B.

Gene ontology (GO) analysis revealed that these three myo-miRNAs are involved in 456 biological processes (BP). Among those, myo-miRNAs were significantly associated with 274 BP, such as ‘muscle contraction’ (33 genes, *p* = 0.012), ‘cellular response to hypoxia’ (32 genes, *p* = 0.05), ‘response to interleukin-1’ (13 genes, *p* = 0.029) and ‘vesicle-mediated transport’ (47 genes, *p* = 0.038), strictly connected with COPD features (Figure 3).

## 4. Discussion

We investigated the expression profile of muscle-specific miRNA in EV isolated from the bloodstream of COPD patients to identify possible differences according to disease severity. We first analyzed total EV content in the plasma of COPD patients and found a significant EV increase in group D patients and a trend towards a positive increase in group B patients, suggesting a correlation between symptom severity and EV [24]. Concerning the primary objective, the results of this pilot study provide three novel pieces of evidence. First, three specific myo-miRNAs, i.e., miR-206, miR-133a-5p and miR-133a-3p.2, were released within EV into the bloodstream of COPD patients; second, EV-derived miR-206, miR-133a-5p and miR-133a-3p were found to be significantly upregulated in GOLD group B compared to groups C, A, and D; third, the triple signature composed of miR-206, miR-133a-5p and miR-133a-3p could help characterize GOLD group B from others.

MiRNAs, and particularly EV-derived miRNAs, have recently emerged in several clinical research areas as a promising novel class of biomarkers able to describe a subset of patients [16,25]. MiR-206, miR-133a-3p and miR-133a-5p, miRNA subgroups in the miR-1 family, are striated muscle-specific miRNA since their expression level is 20-fold higher than their mean expression in other tissues, and they have a significant role during skeletal muscle proliferation, differentiation and regeneration [16,26]. Notably, muscle dysfunction is one of the most relevant systemic manifestations of patients with COPD who experience muscle mass loss or atrophy, especially in the lower limbs [15]. To the best of our knowledge, this is the first report of myo-miRNAs carried in EV in the bloodstream of patients with COPD.

Regarding miR-206, our data are in line with previous evidence from other laboratories showing the upregulation of miR-206 in lung tissues [27], limb muscles [28] and vastus lateralis muscle [29] of patients with COPD compared to healthy subjects, its increased level in the plasma of COPD patients [30] and its inverse association with daily physical activity in the same patients [31]. Further suggestive reports from the literature reveal miR-206 to be upregulated during skeletal muscle regeneration and able to promote differentiation of skeletal muscle satellite cells in vitro [32,33,34]. Accordingly, genetic deletion of miR-206 induced inefficient skeletal muscle regeneration with muscular dysfunction in a mouse model of Duchenne muscular dystrophy [35].

We also show, for the first time, the expression of the two arms of miR-133a (-3p and -5p) in EV isolated from the bloodstream of patients with COPD and also their high expression levels in group B patients. The expression of miR-133a-3p and -5p in circulating EV correlated with their reported expression in plasma [30] and exhaled breath condensates of patients with COPD [36]. Moreover, Donaldson and collaborators reported that plasma-derived miR-133 was higher in patients with the best-preserved lung function [30].

To gain more insight into the potential biological roles played by myo-miRNAs in this particular population, represented by group B COPD patients, in the present study, we conducted a gene ontology analysis, showing a significant association between the biological processes of great interest in COPD, including ‘response to hypoxia’ and ‘muscle contraction’. 

A key original finding is that the panel composed of miR-206, miR-133a-3p and miR-133-5p could help to better characterize GOLD group B patients in relation to others. Indeed, group B represents a challenge: patients belonging to this group, for instance, despite a better lung function, show a worse prognosis over time than group C, probably because they are affected by significant comorbidities [3]. In a post-hoc analysis of a large trial, a group of authors [37] found that group B patients showed a persistent systemic inflammation in their blood over time, and this feature was associated with a worse prognosis during follow-up [38]. Thus, it may be relevant for clinicians to identify biomarkers to distinguish this group from others. In this light, our results, helping differentiate groups B and C, could represent an interesting starting point for further research in this field, even though we are aware that they cannot allow firm conclusions to be drawn, because of the small study sample size and its observational and cross-sectional design; however, our preliminary data suggest that the triple signature of miR-206, miR-133a-3p, and miR-133a-5p could be evaluated in further studies on larger populations as a possible candidate biomarker for identifying group B COPD and differentiating it from other groups. If so, in the future, this panel might hopefully become a tool for monitoring disease progression and perhaps identifying outcomes for therapeutic interventions.

## Figures and Tables

**Table 1:** Main anthropometric and clinical characteristics of the four 2011 Global Initiative for Chronic Obstructive Lung Disease (GOLD) groups.

	Group A (*n* = 4)	Group B (*n* = 9)	Group C (*n* = 8)	Group D (*n* = 14)	*p*-Value
Male/Female	4/0	6/3	8/0	8/6	n.s.
Age (yrs)	74.5 (10.0)	70.0 (12.0)	68 (12.0)	71.5 (3.0)	n.s.
Smoking status (current/former)	1/3	5/4	0/8	3/11	n.s.
Pack-years	39.0 (60.0)	52.0 (18.5)	50.0 (60.5)	48.5 (33.0)	n.s.
Dyspnea, mMRC	1.0 (1.0)	1.0 (1.0)	1.0 (1.0)	2.0 (1.0)	0.002 *
CAT	7.5 (2.5)	11.0 (6.0)	8.0 (9.3)	14.0 (9.5)	0.003 *
BMI, Kg/m^2^	26.7 (10.3)	31.7 (11.4)	29.2 (7.5)	27.4 (7.3)	n.s.
FEV1, L % pred.	1.52 (0.59) 62.5 (30.0)	1.65 (0.53) 62.0 (9.5)	1.37 (0.46) 47.0 (17.3)	0.99 (0.45) 50.0 (16.5)	0.001 ° 0.004 °
FEV1/FVC %	52.0 (17.5)	53.0 (15.5)	42.5 (18.0)	45.1 (16.0)	n.s.
DLCO, mL/min * mmHg % pred.	16.9 (9.7) 84.0 (59.5)	14.0 (8.7) 55.0 (29.5)	24.5 (7.3) 96.5 (33.3)	14.6 (8.6) 59.5 (17.0)	n.s. n.s.
Therapy					n.a.
LAMA			1		
LABA-LAMA	2	4	3	6	
LABA-ICS		1	1	1	
TRIPLE TH.	2	4	2	7	
LABA			1		

* group A vs group D and group C vs group D; ° group B versus group D. n.s.: not significant; n.a.: not applicable.

**Table 2:** Statistical analysis of detectable striated muscle-specific microRNAs from plasma-derived extracellular vesicles and for triple signature in patients with chronic obstructive pulmonary disease (COPD).

EV-Derived Myo-miRna	AUC	95% CI	*p* Value
	GOLD Group B *vs* C		
miR-206	78.89%	63.4–93.7%	<0.0813
miR-133a-5p	75.33%	55.2–88.4%	0.0063
miR-133a-3p.2	73.96%	54.1–89.5%	0.3533
Triple signature	89.74%	70.8–92%	<0.0008
	GOLD Group B *vs* A		
miR-206	78.74%	51.5–90.6%	0.0044
miR-133a-5p	83.82%	52–92.5%	0.0001
miR-133a-3p.2	87.5%	62.5–87.5%	0.1709
Triple signature	83.9%	58.5–91.6%	<0.0001
	GOLD Group B *vs* D		
miR-206	67.09%	49.5–80.2%	0.0134
miR-133a-5p	73.41%	55.7–86.9%	0.0005
miR-133a-3p.2	61.46%	45.2–77%	0.6595
Triple signature	69.4%	53.8–83.4%	<0.0042

## Supplementary Materials

The following are available online at https://www.mdpi.com/2075-4418/10/7/502/s1. Figure S1: ROC curve analysis of the three striated muscle-specific microRNAs (miR-206, miR-133a-5p, and miR-133a-3p.2), which were significantly up—regulated in plasma-derived extracellular vesicles from group B of chronic obstructive pulmonary disease (COPD) patients in comparison with group A, C, and D. ROC, receiver operating characteristic.
