# Supplementary material for: Expression Analysis of Muscle-Specific miRNAs in Plasma-Derived Extracellular Vesicles from Patients with Chronic Obstructive Pulmonary Disease
Source: Diagnostics (Basel). 2020 Jul 21;10(7):502. doi: 10.3390/diagnostics10070502 (PMC7400267; doi:10.3390/diagnostics10070502)

Supplementary Materials:

Figure S1: ROC curve analysis of the three striated muscle-specific microRNAs (miR-206, miR-133a-5p, and miR-133a-3p.2), which were significantly up- regulated in plasma-derived extracellular vesicles from group B of chronic obstructive pulmonary disease (COPD) patients in comparison with group A, C, and D. ROC, receiver operating characteristic.

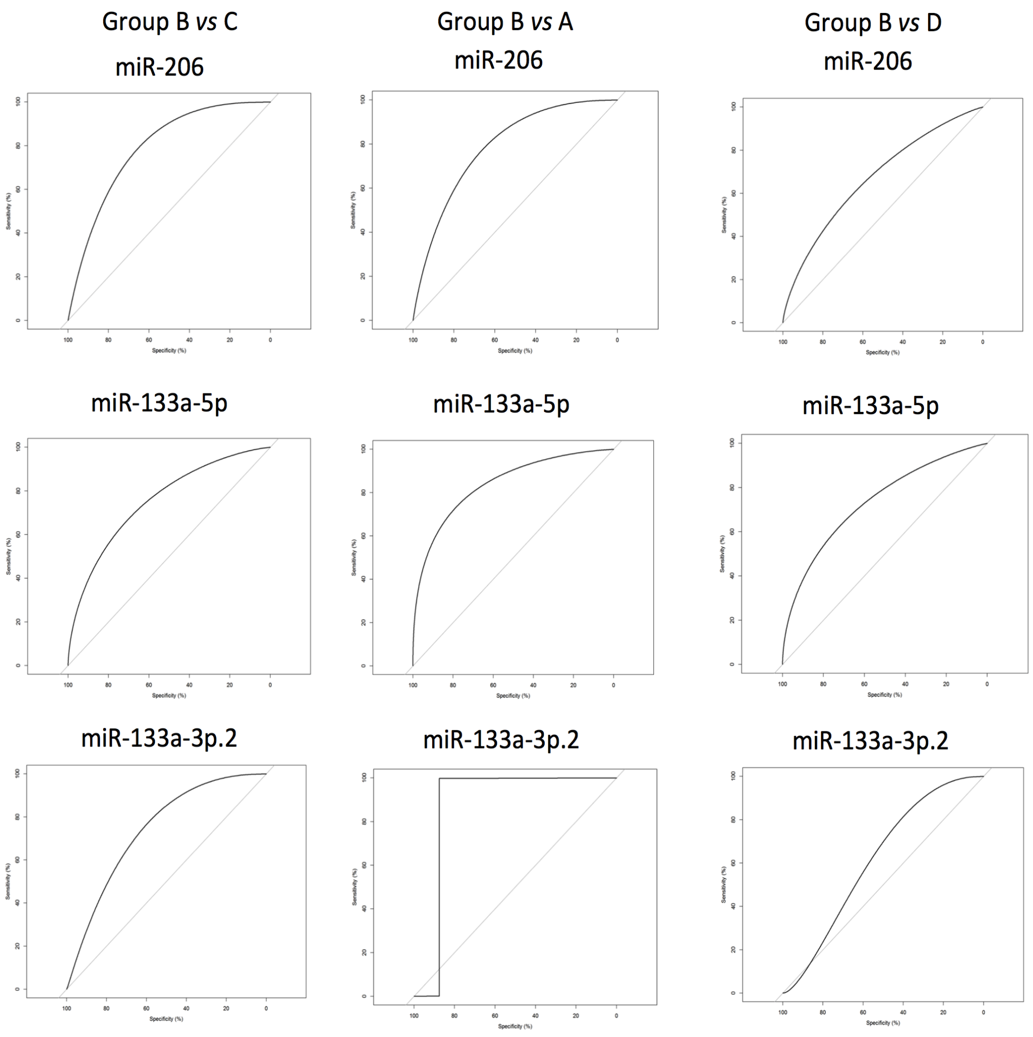

Supplement: Supplementary file 1 [file diagnostics-10-00502-s001.pdf]
